# Nutritional Quality of Breakfast Consumed by the Low-Income Population in Brazil: A Nationwide Cross-Sectional Survey

**DOI:** 10.3390/nu11061418

**Published:** 2019-06-24

**Authors:** Janice Ramos de Sousa, Raquel B. A. Botelho, Rita de Cássia C. A. Akutsu, Renata Puppin Zandonadi

**Affiliations:** Department of Nutrition, Faculty of Health Sciences, University of Brasilia, Brasilia 70910-900, Brazil; janice.rs@hotmail.com (J.R.d.S); rita.akutsu@gmail.com (R.d.C.C.A.A.); renatapz@yahoo.com.br (R.P.Z.)

**Keywords:** breakfast, nutritional composition, low-income, public policies

## Abstract

Objective: This study aimed to characterize the nutritional quality of breakfast consumed by the low-income Brazilian population. Methods: A cross-sectional study was conducted with a sample of 1547 low-income individuals attending 36 Community Restaurants (CRs) in Brazil. Food consumption was analyzed by the frequency of food groups presented in the 24 h recall for three days. The nutritional consumption consisted of the analysis of the total energetic value (TEV) and the macronutrients, fibers, monounsaturated fatty acids, saturated fatty acids, trans fatty acids, calcium, and sodium. The nutrients were evaluated considering the percentage of contribution concerning the daily recommendation of consumption. Results: Among the 4641 breakfasts expected to be consumed and reported, 17.2% (*n* = 797) of the consumers did not consume breakfast. Therefore, we analyzed the 3844 breakfasts. The analysis of food groups showed a high consumption of dairy and cereals, and low consumption of fruits, roots/tubers, and meat/eggs. The percentages of energy contribution of the macronutrients in the TEV were adequate for a balanced diet. The mean intake of monounsaturated fatty acids was low, and the contribution percentages of saturated fatty acids and trans fatty acids were within the recommendations. The mean fiber intake of the breakfast was low, agreeing with the result of low fruit consumption. Calcium intake covered 73.49% of that expected for this meal, and sodium intake was adequate in breakfast. Conclusion: The individuals studied consumed a nutritionally balanced breakfast. Although dairy was one of the most consumed groups, calcium consumption was low, indicating the need to consume higher portions of food sources of this nutrient.

## 1. Introduction

Non-communicable chronic diseases are the leading causes of morbidity and mortality in the world, and unhealthy eating represents one of the main risk factors for such diseases [1]. In Western culture, daily food intake is divided into three main meals: breakfast, lunch, and dinner [2]. Breakfast became important during the industrial revolution as a meal consumed before going to work [2,3], and it has long been described as the most important meal of the day [4]. However, in Brazil, among the three main large meals, breakfast presently tends to be the most impaired meal from the standpoint of consumption [5,6,7]. This feeding behavior is inadequate for human health since there is evidence that breakfast consumption leads to higher nutrient intake and a healthier diet [3,8]. Breakfast is defined as the first meal consumed in the morning [7]. Some studies in Brazil [9,10] state that breakfast consumption should provide 15% to 25% of the daily caloric intake to meet daily requirements. According to some authors [11,12,13,14], a good breakfast should include foods from at least three of the basic groups: dairy, cereals, and fruits; diversifying the consumed food is not only important nutritionally, but it allows breakfast to be more desirable and appetizing. In Brazil, the most frequent breakfast food composition has been pure coffee or milk with coffee, and bread with butter or margarine, and fruit consumption is rare [6]. The last Brazilian Dietary Guideline does not recommend specific quantities of food or nutrients at breakfast; it recommends, according to Brazilian habits, the consumption of natural or minimally processed foods, such as fruits, coffee and milk, and culinary preparations based on cereals, roots, or tubers [15].

Breakfast is important for the achievement of nutrient and health recommendations and has been associated with the intake of most vitamins, minerals [3,8,9,10,11,16], and fibers and a lower intake of fats and cholesterol [17,18]. Studies have shown that its regular consumption is related to health benefits, such as better control of body weight, reduced risk of metabolic syndrome, better functioning of the gastrointestinal system, better mental health; and better performance at both school and work [19,20,21,22]. Many factors influence breakfast consumption, including socioeconomic factors, family structure, and gender [2,3]. Irregular breakfast habits are commonly observed in children and adolescents with low socioeconomic status [4,23], and this irregularity seems to perpetuate into adulthood. However, studies on the adult population are lacking [4]. The present study aims to fill this gap, providing a picture of the Brazilian breakfast consumed by low-income adults. The low-income population, when they do not eat breakfast in their homes, only have access to a precarious meals, opting for foods of low nutritional value and low cost to avoid compromising family income [24].

The Brazilian government has instituted several programs to fight hunger and to improve the food supply of the Brazilian low-income population. Among them, they created the Community Restaurants (CRs) [24,25,26]. This program expands the supply of healthy meals at affordable prices, representing a public policy action focused on reducing the number of people in starving situations via food insecurity and nutritional vulnerability [24,26]. The present research intends to verify the nutritional quality of the breakfast consumed by the low-income Brazilian population in order to contribute to the elaboration and execution of new governmental actions for health promotion. Therefore, this study aims to characterize, nationwide, the nutritional quality of the breakfast consumed by the low-income Brazilian population, according to the criteria adopted by the World Health Organization [27]. Further, we aimed to verify the need to extend the government’s PR program to other meals besides lunch. Therefore, the results of this study will also produce knowledge to evaluate the effectiveness of the Brazilian food and nutrition public policies, which offer only one meal (lunch) to the low-income population in Brazil.

## 2. Material and Methods

This study is exploratory and cross-sectional. The study was approved by the University of Brasília Research Ethics Committee under protocol number 0372/10.

A simple random sample was estimated through the procedure “survey select” of the SAS 9.1.3 program. The final sample included 36 CRs. Based on the total number of users of CRs in Brazil (55,350), we calculated the sample of users to be evaluated for this study considering a 3.5% error and a 95% confidence level. The final sample included 1476 consumers. We chose these local community restaurants because their focus is the low-income population, the target public of this study. The researchers (dietitians) were divided into two groups, with four dietitians in each group, who visited all the restaurants and personally interviewed consumers. They were trained for the interviews with all the materials that the groups took for each CR.

For this study, we chose the 24 h recall to evaluate food consumption. Dietitians conducted the interviews for three days in each CR. In each CR, dietitians gathered information from consumers, writing down all the items and portions cited by each consumer, asking for details on how the food was prepared and the time of each consumption. On Mondays, dietitians got information from Sunday consumption to have one day from the weekend. On Tuesdays and Wednesdays, interviews were conducted to get two more days of 24 h recall (Monday and Tuesday intake). Therefore, the 24 h recall (24 HR) was collected at the CRs for two weekdays and one weekend (via Sunday).

The inclusion criteria were to be a frequent customer (consuming more than three times a week, according to their response during the interview) and over 18 years old. Excluded from the sample were pregnant women, due to their different nutritional needs and individuals under 18 years old. The selection of individuals occurred while they were waiting in line to collect their meals at lunchtime (self-service). It is important to highlight that CRs provide only lunch to the participants. Invitations to participate occurred to the first person in line, then the 15th person, and this pattern was used until the sample was complete. For sampling purposes, users were addressed systematically; that is, 1 out of every 15 who entered the restaurant on the data collection day [28]. They were replaced by the subsequent user when they refused to participate or did not fulfill the assiduity requirement, followed by the 30th, and the 45th, consecutively. 

The breakfast definition for this research was constructed from a preliminary analysis of the daily food consumption report of each participant. Thus, the breakfast meal was defined as the first meal of the day [7], performed from four o’clock in the morning, which may be a single food/preparation or several foods/preparations, and consumed until 11:30 in the morning. This cut-off point in the schedule was also established due to the preliminary analysis of the data, in which the first meal for some participants did not take place until 11:30 a.m., with subsequent consumption of a typical lunch meal. Also, most restaurants start the distribution of lunch at 11:30.

The sociodemographic data collected were sex, age group, income per capita, and education level. All the customers who agreed to participate were invited during one of the days of the research to follow researchers to a private space in the restaurant to take weight and height measurements to classify them according to the Body Mass Index (BMI). The participants were instructed to remove heavy clothing and shoes before being weighed and having their height taken. Body Mass Index (BMI) was used as an anthropometric indicator of nutritional status. Classification occurred according to the criteria adopted by the World Health Organization.

For breakfast evaluation, we classified the food groups. We classified the evaluated food groups as consumed and not consumed, regardless of the size of the portion eaten. All the foods/preparations whose main component is milk were considered as dairy products, such as yogurts, cheeses, vitamins, dairy drinks, ice cream, and porridge. The following cereals were considered as cereals: rice, maize, wheat, morning cereal, granola, cereal bar, oats, and rye. Roots and tubers were considered cassava, manioc flour, potato, Yacon potato, sweet potato, barley potato, yam, and yam since such foods are common in the Brazilian Breakfast.

Although the consumption of meat is not frequent in breakfast, this was evaluated along with the eggs, since the consumption of meat can occur in some regions of Brazil. The nutritional composition of breakfast was evaluated using Dietwin^®^ (Plus version; Porto Alegre, RS, Brazil) software using the Brazilian food composition table database [29]. The data of all the selected individuals were considered valid for the analyses, whether or not they consumed breakfast. For the analysis of the nutritional composition of this meal, the following were evaluated: Total Energy Value (TEV), macronutrient fractions, monounsaturated fat, saturated and trans fats, fiber and micronutrients, calcium, and sodium. Since this study evaluated only breakfast, we did not evaluate the adequacy of consumption, but how much the nutrient consumed in breakfast contributed to the recommendation of each of them.

The TEV of the breakfast meal was evaluated according to Interministerial Ordinance No. 66 [30], which recommends that breakfast be between 300 and 400 calories, which corresponds to 15% to 20% of a 2000 Kcal diet; the percentage of 20% (400 Kcal) was adopted to evaluate energy consumption in this research. Table 1 describes the parameters for the analysis of macronutrients considered in this research.

The calcium and sodium consumption were evaluated according to the recommendations of the Institute of Medicine. We conducted evaluations based on the consumption of the day. The DRI for calcium is 1000 mg/day [33] and, for sodium is 2300 mg/day [34].

Data were tabulated and analyzed in the Statistical Package for the Social Sciences (SPSS®), version 20.0 (IBM Corporation; New York, NY, USA). We counted the number of interviewees, and the number of reminders analyzed, considering the meals performed by the individual. We described data in terms of relative frequencies and summary measures, such as mean and standard deviation. For the association analysis, the statistical test used was Pearson’s chi-square test. The assumptions of normality were checked via the Shapiro-Wilk test. In addition, we used the Student t test and Kruskal-Wallis to verify the analysis of variance, using Tukey as Post-hoc. The significance level adopted was 5%, and the test power was 80% [35].

## 3. Results

Three consecutive days of food recalls were collected from 1547 respondents across Brazil. Of the total of expected breakfast consumption (*n* = 4641) along the three days, 17.2% (*n* = 797) of the days that were analyzed did not contain breakfast; therefore, a total of 3844 breakfast meals were analyzed. Table 2 presents the distribution of sociodemographic variables according to gender. The sample was predominantly composed of men (58.4%, *n* = 903) and individuals aged 25–34 years (men 20.8%, women 21.3%). The most frequent schooling level among the participants was high school education (men 30.8%, women 35.9%), and the most prevalent average income was 0.5 to a minimum wage (MW) (36%) (per capita). Of the population, 64.9% receive an average income up to 1 MW, characterized them as a low-income population. Regarding marital status, there was a predominance of a stable union for males (41.7%, *n* = 377) and a single union for females (37.6%, *n* = 242).

Considering the frequency of breakfast consumption, the mean of breakfast skipping among the three days ranged from 15.7% (*n* = 243) in the Brazilian Northeast region to 21.6% (*n* = 334) in the South region. The average percentage of nationwide low-income breakfast skipping was 17.2%.

For BMI classification, 6.79% of the participants were low weight, 45.44% were normal weight, 33.93% were overweight, and 13.84% were obese. breakfast skipping was not correlated to overweight or obesity (*p* = 0.631; Pearson chi-square).

Table 3 describes the distribution of breakfast food groups by gender, age group, per capita income, educational level, and the geographic housing region of the research participants.

The dairy group was the most consumed during breakfast by both genders (men = 54.3% and women = 61.9%), and the cereals group was the second most consumed (men = 56.5% and women = 58.3%). All the consumers of cereal chose the refined version to consume. Fruits were more commonly consumed in breakfast by males (18.1%, *n* = 163) than females (16.9%, *n* = 109), and the Brazilian region where low-income people most commonly consumed fruit during breakfast was the Northeast region (28.4%, *n* = 108).

The frequency of consumption of roots and tubers (men = 1.8% and women = 2.7) during breakfast was low compared to the other food groups studied. Meat consumption was higher in males (8.4%, *n* = 76) than females. Meat consumption decreased according to the increase in income (income higher than two MW: 5.5%; up to ¼ MW: 12.1%). 

Table 4 presents the national percentages of the contribution of macronutrients to total energetic value (TEV) in the participant’s breakfast. The percentages of energy contribution were adequate, according to the World Health Organization [27]. The percentages of adequacy for the macronutrients were as follows: carbohydrates = 61.1%; proteins = 11.8%, and lipids = 27.3%. We observed that the Southeast region presented the lowest consumption of the breakfast’s TEV (76.2%) compared with the other Brazilian regions. The regional analysis of the mean TEV showed that the North is different from the Northeast (*p* = 0.000), the Northeast is different from the South (*p* = 0.000) and the Southeast (*p* = 0.000), and the Midwest is different from the Southeast (*p* = 0.010) and the South (*p* = 0.044).

The Kruskal-Wallis test showed the statistical difference for the TEV, carbohydrate, protein, and lipid averages. The means of the TEV and carbohydrates consumed were significantly different among the regions (*p* = 0.000 for both), but the mean of the proteins and lipids consumed did not present a statistical difference (*p* = 0.076; and *p* = 0.163, respectively). For the carbohydrates, a difference is found between the North and Northeast regions (*p* = 0.000) and the Northeast and South (*p* = 0.000) and Southeast (*p* = 0.000). However, the Midwest is not statistically different from the other regions (Table 4).

The national consumption of monounsaturated fatty acids was low, and the consumption of saturated fatty acids and trans fatty acids are according to the recommendation. However, analyzing the regions separately, we observed that the percentages of the Midwest region for these same nutrients were high, and the Northeast presented a high percentage of saturated fatty acids. The mean of national fiber intake was 2.1 g ± 1.7 (Table 4), and the North region presented the highest consumption when compared to the other regions.

Although the DRI for calcium is about daily consumption, in Brazil, we consider that breakfast should cover 20% of the daily TEV ingestion. Considering the same percentage, breakfast should cover 200 mg of calcium. Therefore, the national consumption of calcium would be below (147 mg) the expected for this meal.

The DRI for sodium is also considered based on daily consumption, but the application of the reasoning of 20% of breakfast shows that a maximum limit of 460 mg for sodium in breakfast was adequate, since the national consumption was 436.5 mg of sodium (Table 4). 

## 4. Discussion

The evaluated population is representative of CR users, and is considered low income, as the majority receives up to 1 MW, similar to the Brazilian profile.

The percentage of breakfast skipping in the sample studied was lower than that in other Brazilian studies [21,36,37]. The study in [36] that evaluated Brazilian adults (*n* = 600) found that 24% skipped breakfast. Marchioni et al. [21] evaluated 795 adolescents in São Paulo/Brazil and found that 38% skipped breakfast. Eating breakfast is hypothesized to reduce subsequent snack consumption, resulting in lower overall daily energy intake, thus maintaining energy balance and a lower body weight, if it is not associated with higher energy product intake [38]. For low-income people, household food insecurity can limit access to adequate foods, which may lead to problems such as insufficient nutrients and food intake, leading to health issues such as chronic diseases, mental health problems, and poor quality of life [39]. Since skipping breakfast is a risk factor for eating disorders (related to metabolic disorders and depressive symptoms), public policies for the low-income population must be established to improve the health and quality of life of this population [19,20,21,22]. A study [40] showed that the offer of free breakfast for Danish students improved the quality of breakfast intake and reduced breakfast skipping among students at vocational schools. The authors mentioned that free breakfast could be a strategy that authorities might use to improve the eating habits of population groups that are prone to unhealthy eating habits.

The dairy group was the most consumed in both genders (men = 54.3% and women = 61.9%). The high consumption of dairy products was a beneficial factor in the studied sample because it is this group’s source of protein and calcium, essential nutrients for several biological functions [41]. This finding is consistent with the results of other studies, such as the study carried out by Durá Travé [11], who nutritionally analyzed the consumption of CM and lunch of 740 Spanish university students. The result of this study showed that the most consumed groups in the CM of the participants were dairy (92.6%) and cereals (58.8%). In the research conducted by Marchioni et al., [21], for example, whose purpose was to characterize the standards and nutritional contribution of the CM of 795 Brazilian adolescents from São Paulo, the milk and dairy groups represented the second most consumed. It is interesting to emphasize that even though the population is low income, they still acquire and use dairy products that are usually more expensive products.

The cereal group was the second most consumed (men = 56.5% and women = 58.3%) when the gender variable was evaluated. We expected such results, since refined cereals are the basis of low-income diets, with low cost and easy and long storage [26,42,43]. Souza et al. [8], when describing the food and macronutrient intake of 71,791 Brazilian adolescents from a national school-based survey, found that foods with a higher prevalence of consumption included cereal-based products, such as rice (82%) and bread (53%).

Studies suggest that a standard composition for breakfast should contain at least three basic food groups: dairy, cereals, and fruits [11,44]. On the other hand, some authors state that socioeconomic factors, such as educational level, family income, gender, and housing region, are essential determinants of food choice [37,45,46], impacting the composition of the breakfast consumed. In Brazil, there is a higher daily intake of animal protein and lower carbohydrate consumption with increased income [47]. The trend of dairy and cereal consumption [47] was similar to our findings. In the present study, although dairy products presented no significant difference (*p* = 0.89), there was lower consumption in the income range up to ¼ MW (50.3%) and higher in the income range above 2 MW (61%). In contrast, refined cereals had higher consumption in the income range up to ¼ MW (57.8%) and lower consumption in the group with more than 2 MW (55.9%), with a significant difference (*p* = 0.03).

Although not significant (*p* = 0.122), there is a higher fruit consumption by males (18.1%, *n* = 163) than females (16.9%, *n* = 109), different from most data found in national surveys [45,47,48,49]. In the study conducted by Neutzling et al., [45], whose objective was to describe the frequency of consumption of fruits and vegetables by 972 adults living in Pelotas-RS/Brazil, from 20.9% interviewed who reported regular consumption of fruit, 26.9% were women and (12.9%) were men. Mondini et al., [32] evaluated the consumption of fruits and vegetables by 930 adults in the city of Ribeirão Preto-SP/Brazil, in which only 24% of men and 38% of women met the minimum recommended consumption of fruits and vegetables. Charlton et al., [50] found that Australian men eat fewer fruits and vegetables than women (*p* < 0.0001). Among Chilean adults, Pienovi et al., [51] did not find a significant difference in fruit and vegetable intake between the genders (*p* = 0.154).

The participation of fruits and vegetables in the Brazilian diet is low [21,52]. Data from the Brazilian Family Budget Survey [53] showed that fewer than 10% of Brazilians reached the recommended portions for fruits and vegetables. This group contributes only with 2.8% of the daily total energetic value [53,54]. Scientific evidence suggests that fruit and vegetable consumption plays a protective role against non-transmissible chronic diseases due to their content of micronutrients, antioxidants, phytochemical compounds, and fiber [9,50,51,55,56]. Therefore, fruit and vegetable consumption should be higher than we found in the present study (for both genders), starting with the first meal of the day, since breakfast is a fundamental requirement for the best compliance with the daily recommendation of 400 g of fruits and vegetables [9,56].

As expected, fruit consumption in the present study was higher in the higher income stratum (>2 MW = 23.6%). This result is confirmed by the analysis of the Brazilian family budget survey [53], which indicates that fruits are one of the food groups in which participation in daily consumption tends to increase with the level of family income increasing. This is probably because diets high in fruits and vegetables are more expensive than others. Mondini et al. [48], evaluating the consumption of fruits and vegetables in 930 adults from Ribeirão Preto-SP/Brazil, observed the same association of an increase in fruit consumption with the increase in per capita income and schooling. The existence of economic restrictions on the value of food leads to diets with low participation of fruits and vegetables and with high energy density [57,58]. breakfast composition can impact the body mass index and the risk of developing noncommunicable diseases [4,38]. The combination of whole cereals with dairy products plays a protecting role, while high-fat and processed foods, such as processed meat, cheese, and margarine, are associated with a higher risk of developing noncommunicable diseases [4]. It is important to highlight that in the present study, although the analysis of the diet detected a low consumption of fruits and vegetables, the consumed breakfast meal did not present, on average, a high energy density. 

The low frequency of root and tuber consumption in breakfast is justified by the fact that this kind of food is not usually consumed in all regions of Brazil. In general, only the Northeast and North Brazilian regions population have the habit of consuming roots and tubers for breakfast [59,60,61,62,63]. However, our result is in accordance with the results [64] from another study that evaluated 7425 Brazilian adolescents (data from the national survey “Family Budget Survey” [53]), in which the frequency of consumption of roots and tubers in breakfast was 2%. The consumption of roots and tubers in substitution of a bakery in breakfast could improve the nutritional quality of the Brazilian population’s diet, since they are sources of carbohydrates, fibers, minerals, and vitamins. Also, the substitution of baked goods for roots and tubers can reduce sodium ingestion.

The highest consumption of meat by men (8.4%, *n* = 76) was also consistent with the data from the Brazilian survey on Risk Factors and Protection for Chronic Diseases by Telephone Inquiry (VIGITEL) [60]. The survey evaluated more than 52 thousand adults in the Brazilian population (benefiting from health insurance plans) and showed that 26.5% of them consume high-fat meat. The consumption was higher by men (35.2%) than by women (19.5%). It is important to highlight that VIGITEL [60] showed that the frequency of consumption of meat with excess fat tended to decrease with an increase of the age group, with no difference for schooling, as we also observed in the present research.

In the present study, the majority (participants from 29 CR) consumed breakfast at home. The interviewed individuals consumed a balanced breakfast based on the distribution of macronutrients, regardless of their sociodemographic conditions. The study conducted by Araújo et al., [65] with 51 users of the Community Restaurant in Fortaleza/Brazil, also showed a total energetic value (TEV) adequacy and balanced distribution of macronutrients at lunch. Although the study mentioned above focused on lunch, unlike the present study that studied breakfast, this finding indicates that the low-income population that uses CRs is more concerned about healthy eating [25,26].

The low national average consumption of monounsaturated fatty acids in breakfast meals is not considered a relevant aspect, since the food sources of such nutrients [6], except avocado, are not commonly consumed in Brazilian breakfast. In the present study, participants consumed adequate amounts of saturated fat for breakfast. The study conducted by Araújo et al., [65] with CR users, showed the same result regarding the saturated fat consumption profile. Even though their results were related to the lunch meal, it is important to compare since the studies evaluated a similar population, and also because of the lack of Brazilian studies on breakfast in a low-income population.

Marchioni et al. [21] considered that almost 40% of the daily consumption of trans fat occurs in breakfast. In this sense, it can be inferred that the low consumption of this nutrient in most of the Brazilian regions was a satisfactory result in the diet profile of individuals. However, fiber intake was lower than the recommendation by the American Dietetic Association (14 g dietary fiber per 1000 kcal, or 25 g for adult women and 38 g for adult men) [32], which is consistent with the low fruit consumption by the participants of the present study and their preference for refined cereals (Table 3).

Studies have shown that adequate fiber consumption can be a protective factor for heart disease, cancers, diabetes, and gastrointestinal disorders, specifically by maintaining low blood levels of cholesterol, normalizing serum glucose and insulin levels, and improving intestinal transit lower risk for colon cancer [32,57,66,67]. Therefore, fruit consumption can contribute to the prevention of chronic non-communicable diseases, which represent the primary cause of death in Brazil and worldwide [1].

Although the national consumption of calcium (147 mg) reached 73.49% of the expected level for this meal, in the Brazilian breakfast, we expected that calcium consumption would be higher than in other meals, since Brazilian breakfast tends to include dairy products [7]. Considering that the consumption of calcium prevents diseases like osteoporosis, arterial hypertension, colon cancer, and obesity [41,68], and that the only source of calcium available to humans is from their diet, it is essential to ensure intake of this mineral [68]. Therefore, the calcium ingestion by our study participants could be higher than we found, although the dairy group represents one of their most consumed groups during breakfast. It is important to mention that increasing dairy product portions would mean an increase in the cost of breakfast. Therefore, it is important that low-income participants consume other sources of calcium in the lunch meals provided by the CR program. Alternatively, the government could invest in the supply of breakfast in these CRs.

A study that evaluated the sodium consumption by 22,279 Brazilians showed that the mean sodium consumption at breakfast was 422 mg, similar to the results found in our study (436.5 mg of sodium) [69]. The same authors created the “breakfast quality index” for the Brazilian population, and they considered the following as sodium parameters for breakfast: low (398 mg), medium (427 mg), and high (464 mg). Since the DRI for sodium is considered based on daily consumption, if breakfast represents 20% of the daily intake, the maximum sodium maximum should be 460 mg, a limit similar to the one proposed by Lopes Pereira et al. [69] as high sodium consumption. In this sense, the low-income participants in our study seem to consume an amount of sodium during breakfast similar to the general Brazilian population. Although the national consumption of sodium is adequate, it is necessary to mention that bread is one of the main contributors to sodium intake during breakfast in Brazil. Also, consumption of foods like sausages and some types of cheese also influences sodium intake and needs to be monitored to achieve a healthy diet. 

In the present study, skipping breakfast was not correlated to overweight or obesity (*p* = 0.631), thereby corroborating Betts et al. [70], who did not find a difference between the body mass of breakfast eaters and skippers. Studies showed that skipping breakfast was correlated to obesity [71,72,73]. However, the population studied was different from ours, as our study evaluated the low-income Brazilian population using the CRs as the main source of food at lunch. This population, in general, did not have access to buy snacks to consume all day. Studies showed that eating breakfast in the morning reduces the number of meals (avoiding snacks), leading to a reduction of BMI [44,74]. In the population of our study, however, the consumption of breakfast probably does not influence the consumption of snacks due to a lower access to buy foods (snacks). 

## 5. Conclusions

This is the first study that nationally evaluates the breakfast consumed by low-income Brazilians. The analysis of food groups shows high consumption of dairy and cereals products, and low consumption of fruits, roots/tubers, and meat/eggs. Men consumed more fruits, especially men from 35 to 44 years old and with the highest income. Dairy and cereals products had an opposite trend of consumption when income was considered. The national percentages of the energy contribution of the macronutrients in the TEV, as well as the analysis of fatty acids, were within the recommendation. The national average of breakfast fiber intake was low, agreeing with the result of low fruit consumption, and consumption of more refined cereals, usually cheaper and with better sensorial acceptability. Skipping of breakfast was not associated with overweight or obese participants. There is a consensus on the need for quality breakfast choices with high nutritional value to improve health outcomes. Considering the low consumption of fruits and vegetables by low-income Brazilians associated with the cost of this type of food, it is important to evaluate if it is necessary to expand the Brazilian social program of CRs to provide more than lunch meals as a benefit for the low-income population’s health. Further studies are necessary to evaluate whether the content, timing, or regularity of breakfast intake is associated with lower chronic diseases risk in the low-income Brazilian population. It is also important.

## Figures and Tables

**Table 1:** Distribution of macronutrient recommendations on total energetic value (TEV) of 400 kcal of breakfast.

Nutrient	Reccomendation (%)	Reccomendation (Kcal)	Reccomendation (g)
Carbohydrates	55 to 75 [27]	220 to 300	55 to 75
Proteins	10 to 15 [27]	40 to 60	10 to 15
Lipids	15 to 30 [27]	60 to 120	6.7 to 13.3
Monounsaturated fatty acids	10 to 15 [31]	40 to 60	4.4 to 6.7
Saturated fatty acids	<10 [31]	40	<4.4
Trans fatty acids	<1 [27]	4	<0.4
Fiber	10 to 14 g/1000 Kcal [32]		4.0 to 5.6

**Table 2:** Distribution of sociodemographic data frequency among Community Restaurants (CRs) users regarding breakfast consumption.

Variables		Male		Female		Total	
		N	%	N	%	N	%
Gender		903	58.4	644	41.6	1547	100
Age (years)	18–24	94	10.4	96	14.9	190	12.3
	25–34	188	20.8	137	21.3	325	21
	35–44	150	16.6	109	16.9	259	16.7
	45–54	168	18.6	105	16.3	273	17.6
	55–64	131	14.5	82	12.7	213	13.8
	>65	172	19	15	17.9	287	18.6
Income (*per capita-minimum wage)*	Up to ¼ MW	64	7.1	69	10.7	133	8.6
	¼–½ MW	170	18.8	145	22.5	315	20.4
	½–1 MW	324	35.9	232	36	556	35.9
	1–2 MW	201	22.3	125	19.4	326	21.1
	>2 MW	85	9.4	42	6.5	127	8.2
	Without information	59	6.5	31	4.8	90	5.8
Educational level	Illiterate	36	4	21	3.3	57	3.7
	IPE	274	30.3	182	28.3	456	29.5
	CPE	129	14.3	78	12.1	207	13.4
	IH	60	6.6	36	5.6	96	6.2
	CH	278	30.8	231	35.9	509	32.9
	TC	7	0.8	10	1.6	15	1.1
	IUG	54	60.2	34	5.3	88	5.7
	CUG	65	7.2	52	8.1	117	7.6
Marital status	Single	353	39.1	242	3.6	595	38.5
	With Partner	377	41.7	241	37.4	618	39.9
	Widow	51	5.6	78	12.1	129	8.3
	Divorced	122	13.5	83	12.9	205	13.3

IPE: Incomplete Primary Education; CPE: Complete Primary Education; IH: Incomplete high school; CH: Complete High School; TC: Technician course; IUG: Incomplete Under-graduate; CUG: Complete Under-graduate; Minimum wage (MW): USD $175.86 per month.

**Table 3:** Distribution of breakfast food groups by gender, age group, per capita income, educational level, and geographic housing region of the research participants.

Variables		*n*	%	Food Groups (%)				
				Dairy Products	Cereals	Fruits	Roots and Tubers	Meat or Eggs
**Gender**	Male	903	58.4	54.3	56.5	18.1	1.8	8.4
	Female	644	41.6	61.9	58.3	16.9	2.7	5.4
**Age (year)**	18–24	190	12.3	53.1	48.4	14.2	3.1	7.1
	25–34	325	21	55.1	56.4	16.3	1.6	8.4
	35–44	259	16.7	55.8	56.7	20.3	2.1	9.3
	45–54	273	17.6	53.8	55.1	16.1	1.6	7.5
	55–64	213	13.8	57.2	57.5	19	2.3	4.9
	>65	287	18.6	68.3	66.2	19.1	2.6	5.1
**Income** ***per capita (minimum wage)***	Até ¼ MW	133	8.6	50.3	57.8	16.5	0.7	12.1
	¼–½ MW	315	20.4	55.7	54.3	17.4	4	6.2
	½–1 MW	556	35.9	57.8	59.2	15.3	1.7	7.3
	1–2 MW	326	21.1	61.6	57.8	13	1.8	6.7
	>2 MW	127	8.2	61	55.9	23.6	1.9	5.5
	Without information	90	5.8	52.2	53.9	18.3	2.2	6.1
**Educational level**	Illiterate	57	3.7	54.4	70.2	3.5	0	3.5
	IPE	456	29.5	53.1	75.9	10.1	0.2	5.3
	CPE	207	13.4	54.1	82.1	10.6	2.4	4.8
	IH	96	6.2	56.3	79.2	16.7	3.1	3.1
	CH	509	32.9	56.6	79.6	12	1.2	4.1
	TC	17	1.1	41.2	76.5	17.6	0	0
	IUG	88	5.7	50	78.4	17	0	3.4
	CUG	117	7.6	57.3	82.1	15.4	1.7	0.9
**Housing geographic regon**	Midwest	43	2.8	65.1	62.8	16.3	1.1	12.8
	Northeast	381	24.6	47.8	48.8	28.4	3.8	13.6
	North	175	11.3	68.8	70.6	12	0.2	6.8
	Southeast	645	41.7	61	59.2	13.9	1.1	3.5
	South	303	19.6	54.6	55.3	15	2.5	6.2
	Brazil	1547	100	59.5	59.3	17.1	1.7	8.6

IPE: Incomplete Primary Education; CPE: Complete Primary Education; IH: Incomplete high school; CH: Complete High School; TC: Technician course; IUG: Incomplete Under-graduate; CUG: Complete Under-graduate; Minimum wage (MW): USD $175.86 per month.

**Table 4:** Mean and standard deviation of the low-income population’s ingestion of nutrients during breakfast by Brazilian region.

Nutrient		Brazil (*n* = 1.491)	Midwest (*n* = 40)	Nordtheast (*n* = 377)	North (*n* = 168)	Southeast (*n* = 624)	South (*n* = 282)
TEV (Kcal)		340.5 ± 201.7	400.9 ± 207.2	405.8 ± 225.5	335.8 ± 208.1	304.9 ± 182.2	326,59 ± 182,45
Carbohydrates (g)		52 ± 29.8	57.6 ± 32,55	63 ± 34.9	51.2 ± 27.9	46.5 ± 25.9	49.2 ± 27.4
Proteins (g)		10 ± 8	10.8 ± 6.7	11.8 ± 8.9	9.3 ± 10.2	9.2 ± 6.2	10 ± 8.4
Lipids (g)		10.3 ± 8.8	14.4 ± 8.6	11.8 ± 9.6	10.3 ± 8.1	9.3 ± 8.3	10 ± 9
Monounsaturated fat (g)		2 ± 2.3	3 ± 2.5	2.1 ± 2.4	2 ± 2.2	1.8 ± 2.1	2.1 ± 2.8
Saturated fat (g)		3.8 ± 3.4	5.9 ± 3.8	4.6 ± 3.8	3.8 ± 3.7	3,3 ± 2.7	3.3 ± 3.5
Trans fat (g)		0.2 ± 0.6	0.5 ± 0.9	0.3 ± 0.7	0.3 ± 0.7	0.2 ± 0.61	0.2 ± 0.5
Fiber (g)		2 ± 1.7	1.8 ± 1.1	2.6 ± 1.7	2.8 ± 2.2	1.6 ± 1.5	1.9 ± 1.5
Calcium (mg)		147 ± 134.5	187.2 ± 166.1	132.1 ± 118.7	143.8 ± 132.2	152.6 ± 141.5	150.4 ± 133.4
Sodium (mg)		436.5 ± 376	682.2 ± 477.6	562.2 ± 495.2	384.7 ± 306.5	386 ± 312.6	376.6 ± 277.1
***Contribition (%)***							
Nutrient	Reccomendation						
TEV	400 Kcal	85.1	100.2	101.5	84	76.2	81.7
Carbohydrates	75 g [27] *	69.4	76.8	84	68.3	62	65.7
Proteins	15 g [27] *	67.1	67.1	78.4	62.2	61.6	66.6
Lipids	13.3 g [27] *	77.7	108.3	89	77.4	70.1	75.1
Monounsaturated fat	6.7 g [31] *	29.7	44.8	31.6	30.2	26.7	31.2
Saturated fat	<4.4 g [31]	85.2	134.1	103.6	86.4	75.2	75.2
Trans fat	<0.4 g [27]	60	125	80	67.5	50	43
Fiber	5.6 g [32]	36.6	31.6	46.1	50.2	28.4	34.6
Calcium	1000mg/day [33] **	14.7	18.7	13.2	14.4	15.3	15
Sodium	2300mg/day [34] **	19	29.7	24.4	16.7	16.8	16.4

TEV: total energetic value. * We considered the upper recommendation limit. ** Contribution percentage related to the daily consumption recommendation.
